# Normative values and psychometric properties of the Oslo Social Support Scale-3 (OSSS-3) for adults aged 60 to 85 years

**DOI:** 10.1007/s10433-025-00867-9

**Published:** 2025-07-08

**Authors:** Nikolay Dimitrov, Elmar Brähler, Thomas Hering, Heide Glaesmer, Markus Zenger

**Affiliations:** 1 Department of Applied Human Studies, University of Applied Sciences Magdeburg-Stendal, Osterburger Str. 25, 39576 Stendal, Germany; 2 Department of Psychosomatic Medicine and Psychotherapy, University Medical Center of the Johannes Gutenberg-University Mainz, Mainz, Germany; 3 Department of Medical Psychology and Medical Sociology, Leipzig University Medical Center, Leipzig, Germany; 4 Integrated Research and Treatment Center Adiposity Diseases, Behavioral Medicine Unit, Department of Psychosomatic Medicine and Psychotherapy, Leipzig University Medical Center, Leipzig, Germany

**Keywords:** Oslo Social Support Scale-3 (OSSS-3), Social support, Older adults, Normative values, Psychometric properties, Healthy aging

## Abstract

The main objective of the current study was to investigate the psychometric properties of the Oslo Social Support Scale (OSSS-3) and establish detailed normative values for older adults aged between 60 and 85 years. The representative sample analyzed consists of German residents aged between 60 and 85 living in private households (N = 1659). The analysis of the psychometric properties of the OSSS-3 involved reliability and validity testing as well as an EFA and a CFA. We provide age-specific normative data for the OSSS-3 for the German population aged between 60 and 85. The EFA resulted in a one-factor model for OSSS-3, and the CFA confirmed that this model fits the data well. In accordance with previous studies on this topic, we found that the OSSS-3 is a reliable and valid instrument for assessing perceived levels of social support among older adults. The generated percentile norms allow the direct comparison of individual scores of older adults on the OSSS-3 to an age-corresponding reference sample. Exploring the levels of perceived social support among older adults is important, given the low levels of perceived social support experienced by approximately 25% of older adults in Germany. The risk factors for social isolation and its consequences for the mental and physical health of older adults are discussed. If the population continues to age as expected, an even greater number of older adults in the future could face low levels of perceived social support.

**Supplementary Information:**

The online version contains supplementary material available at 10.1007/s10433-025-00867-9.

## Introduction

Increased life expectancy and declining birth rates are leading to dramatic demographic changes worldwide. The absolute number and proportion of people over 60 years of age have grown rapidly in the last century and are projected to grow even faster in the twenty-first century (Gu et al. 2021). The increasing number of older adults adds to the importance of detailed normative values for constructs relevant to their age. The current study addresses this need by investigating the psychometric properties of the Oslo Social Support Scale (OSSS-3) and providing normative values for the German population aged 60 to 85 years.

The added years of life could allow the older generation to pursue new roles in society, new ways to contribute and thus ‘age successfully’ (Rowe & Kahn 1997). In reality, despite life expectancy growth, the absolute number of years in good health has remained constant (Abbafati et al. 2020). The added years of life expectancy are often experienced in suboptimal physical health. To maximize the benefits and manage the risks associated with population aging, supporting healthy aging among older adults should become a high-priority task of our society.

The term ‘healthy aging’ is relatively novel, but it is being actively researched (Michel & Sadana 2017), and its importance is already being recognized by the World Health Organization (WHO), as they announced the current decade (2021–2030) as the ‘United Nations’ Decade of Healthy Ageing’ (Rudnicka et al. 2020). The WHO's definition of healthy aging goes beyond the traditional view of health as the bare absence of disease and defines ‘healthy aging’ in a more holistic manner as ‘the process of developing and maintaining functional ability that enables well-being in older age’ (World Health Organization 2015).

Social support is being recognized as one of the most important determinants of healthy aging (Vila 2021). There are several ways in which sufficient levels of social support could be contributing to the process of healthy aging by older adults. High levels of social support slow cognitive aging in healthy older adults (Seeman et al. 2001). It also stimulates exercise behavior, which is highly beneficial for the older generation (Resnick et al. 2002). Older adults receiving sufficient levels of social support are less likely to suffer from depression (Schwarzbach et al. 2014) and self-report better health status (White et al. 2009). High levels of social support have also been found to be a predictor of happiness (Ahmed & Mohamed 2022), are associated with lower levels of mortality and morbidity (Berkman 1985), and are important predictors of the well-being of older adults (Y. Chen & Feeley 2014; Krause 1986).

Social support refers to the availability of assistance and protection in various forms that are expected to be provided by a person’s network of relationships when needed (Langford et al. 1997). Despite the extensive research on the topic, there is still a lack of uniform agreement in the literature about its exact conceptualization (Chappell & Funk 2011; Langford et al. 1997). Early on, some authors noted as a possible reason for this ambiguity the multimodality of the concept that led to too many definitions and approaches of measure (Barrera 1986).

Two dimensions of social support are widely recognized: structural (Seeman & Berkman 1988) and functional (Semmer et al. 2008). While structural social support refers to the quantity and composition of social relationships, such as size, diversity and overall social integration of the individual’s social network, functional social support refers to the specific type and quality of received social support. Functional social support can be further divided into different categories, such as emotional, instrumental, informative, appraisal and companionship support.

A distinction is also made between received social support, which refers to concrete supportive behaviors provided by others, and perceived social support, which reflects an individual’s general sense of available or satisfactory support (Sarason et al. 1990). Received support is considered to better reflect actual external support due to its focus on observable behaviors (Barrera 1986), whereas perceived support is more susceptible to subjective interpretation (Lakey & Drew 1997). Although the two constructs are moderately correlated (*r* = 0.35; Haber et al. 2007), perceived support, despite its inherent subjectivity, is regarded as the most important dimension of social support with respect to health outcomes (Uchino 2009). As a multidimensional construct, there are several different approaches for measuring social support. Some of them assess social support as a global concept, whereas others assess only its structure or concrete function (Gottlieb & Bergen 2010).

The current study aims to provide normative data for older adults for the OSSS-3, which measures the level of perceived social support. Information about the origins of the OSSS-3 is limited. The scale was first developed by Dalgard (1996), but the original publication is not available online and is overall hard to acquire. The OSSS-3 gained wider recognition when it was selected as a measure of perceived social support in the EUROHIS study (Buratta et al. 2003), and it has since been employed in numerous large-scale studies such as the European KIDSCREEN Study (Ravens‐Sieberer et al. 2008) and the Outcome of Depression European Network study (Dowrick et al. 1998).

Its widespread application across all age groups can be attributed to its simplicity, brevity and strong face validity. The importance of perceived social support as a construct grew rapidly during the lockdowns that emerged in several countries during the COVID-19 pandemic. The OSSS-3 has been utilized to assess social support in several studies on the various effects of the pandemic on the psychological and physical well-being of the population (Faris et al. 2023; Sehlo et al. 2022; Yao 2020). Several studies have measured the levels of social support of older adults with the OSSS-3 for various topics (e.g., Nemcikova et al. 2023; Sánchez-Moreno & Gallardo-Peralta 2022), but until now, no normative values for older adults have been published.

The validity of the OSSS-3 has been demonstrated in three studies so far (Bøen et al. 2012; Glaesmer et al. 2011; Kocalevent et al. 2018). Kocalevent et al. (2018) also tested the scale's reliability, researched the factor structure of the OSSS-3 and provided normative values for the general German population. However, the norms for the older generation are given in age groups that are too broad. There are only three age brackets for adults over 55 years old. As this instrument could be valuable for researching social support by older adults, finer normative values would be beneficial. The OSSS-3 is a one-dimensional self-administered instrument that is brief and simple. It consists of only three easily accessible items. OSSS-3 is best suited for large-scale research projects, as it adds a minimum amount of time and effort from the participants. The three questions of the OSSS-3 gather information about the number of close people if the people in their lives are concerned with and interested in them and if it is easy to receive practical help from neighbors when needed.

## Methods

### Study sample

In 2008, a two-stage nationwide study in Germany was conducted in collaboration with USUMA (Berlin), an institute specializing in demographic research. Two separate samples were collected in two stages. In the first stage, the sample gathered was representative of the general German population, and in the second stage, it was representative of the German population aged 60–85 years. The study adhered to the regulations outlined in the German data protection law (§30a BDSG) and was conducted according to the principles established by the Declaration of Helsinki.

The sample analyzed within the current study consists of German residents aged between 60 and 85 years of age, derived from both stages, who were living in private households (*N* = 1659). Each of the participants was informed about the study and signed an informed consent. The age, gender and educational attainment of the participants were matched with the records of the Federal Elections registry, ensuring that the sample was representative. Two callback efforts were made before an address was classified as unattainable. The sampling methodology consisted of a multistage process involving sample points, households and individual participants in the final stage. Target households within the sample points were identified via a random-route procedure, which included selecting sample point areas across Germany, random household selection within these areas and the random choice of specific individuals within these households. Within the broader survey framework, study participants were interviewed via a structured self-report questionnaire, which included the OSSS-3.

### The instrument: oslo social support scale (OSSS-3)

The OSSS-3 consists of three items measuring the perceived level of social support. The three items of the OSSS-3 and the respective possible choices are as follows:

Oslo 1: How many people are so close to you that you can count on them if you have great personal problems?

1 ‘none’.

2 ‘1–2’

3 ‘3–5’

4 ‘5 + ’.

Oslo 2: How much interest and concern do people show in what you do?

1 ‘none’.

2 ‘little’.

3 ‘uncertain’.

4 ‘some’.

5 ‘a lot’.

Oslo 3: How easy is it to get practical help from neighbors if you should need it?

1 ‘very difficult’.

2 ‘difficult’.

3 ‘possible’.

4 ‘easy’.

5 ‘very easy’.

The test score is the sum of the scores of the three items. It ranges from 3 to 14, with low values indicating low levels of perceived social support and high values indicating strong levels of social support. The sum score was used to calculate the norm values for the different age groups, as well as to test for group differences. The standard method of obtaining percentile ranks was applied (Crawford et al. 2009).

The OSSS-3 scores are often categorized into three broad, non-validated levels of perceived social support, as suggested by Bøen et al. (2012): from 3 to 8, poor social support; from 9 to 11, moderate levels of social support; and from 12 to 14, strong levels of social support.

## Data analysis

Two separate one-way analyses of variance (ANOVAs) were conducted with gender and age as between-subject factors. Tukey's honest significant difference test was performed as a post hoc test when significant group differences were found. The construct reliability of the OSSS-3 was tested through its internal reliability, measured with Cronbach’s alpha and McDonald’s omega.

The factor structure of the OSSS-3 among older adults was tested with exploratory factor analysis (EFA). The data from all the participants were split into two halves randomly: One was used for the EFA, and the other was used for the confirmatory factor analysis (CFA). Principal component analysis (PCA) without rotation was the main chosen method. A rotation was not undertaken because of the small number of items. The number of factors extracted was initially based on the eigenvalue. Every factor with an eigenvalue over 1.0 was retained. A scree plot was also examined. Additionally, to determine the optimal number of factors to retain, Horn's *parallel analysis (*PA*,* Horn 1965) was utilized*.*

To test the validity of the factor solution found by the EFA, a CFA was conducted with the remaining half of the data. Various model fit indices and their corresponding criteria were employed to evaluate the adequacy of the model. Among these indices were the goodness-of-fit index (GFI), the comparative fit index (CFI), the Tucker‒Lewis index (TLI), the standardized root-mean-square residual (SRMR) and the root-mean-square error of approximation (RMSEA).

Due to the limited number of additional questionnaires administered alongside the OSSS-3, no alternative measure of perceived or received social support was available in the dataset. As a result, it was not possible to assess convergent validity. Instead, we aimed to explore the divergent validity of the OSSS-3.

The chosen instruments were the Patient Health Questionnaire-9 (PHQ-9, Martin et al. 2006), the Patient Health Questionnaire-15 (PHQ-15, Kocalevent et al. 2013), the Posttraumatic Diagnostic Scale (PDS-5, Foa et al. 2016) and the 12-Item Short Form Survey (SF-12, Drixler et al. 2020).

Individuals with lower levels of perceived social support are more likely to report higher levels of depressive and somatic symptoms (Grigaitytė & Söderberg 2021; Wang et al. 2018). However, since these instruments assess mental and physical distress rather than perceived social support directly, only weak correlations between the OSSS-3 and both the PHQ-9 and PHQ-15 were anticipated.

Similarly, we expected weak to very weak negative correlations between the OSSS-3 and the PDS-5, which assesses posttraumatic stress symptoms. Although traumatic stress and social functioning are closely interrelated, PTSD and perceived social support are conceptually distinct constructs, and previous studies have demonstrated only limited associations between them (Robinaugh et al. 2011).

Finally, we anticipated weak to very weak correlations between the OSSS-3 and the SF-12, a widely used measure of self-reported health-related quality of life, given that perceived social support and general health perceptions are related but separate domains (Yoo et al. 2017).

The statistical analyses were conducted via SPSS 29 and AMOS 29, with an α-level of 5%.

## Results

### Sample characteristics

The study sample consisted of 1659 adults aged between 60 and 85 years. Five participants were removed from further analysis because of a missing response to at least one of the OSSS-3 items; thus, *N* = 1654 participants (916 women) were further analyzed. The demographic characteristics of the sample are presented in Table 1. Overall, men and women differ significantly from one another in various demographic characteristics. There were more women than men in the older age groups (χ2 = 20.33, *p* < 0.001) and more women reported living alone (χ2 = 179.903, *p* < 0.001) and being widowed (χ2 = 234.48, *p* < 0.001). Women are also more likely to have a lower educational status (χ2 = 19.55, *p* < 0.01).

**Table 1:** Demographic characteristics of the sample

Variables	*n*	%
*Sex*		
Male	738	44.6
Female	916	55.4
*Age group, years*		
60–64	370	22.4
65–69	465	28.1
70–74	340	20.5
75–79	263	15.9
80–85	216	13.1
*Marital status*		
Married/Living together	1099	66.5
Married/Living separated	5	0.3
Single	40	2.4
Divorced	77	4.6
Widowed	433	26.2
*Net income*		
Not specified	55	3.3
< 1250€	1074	65
1250—< 2500€	481	29.1
> 2500€	44	2.6
*Employment*		
Yes	107	6.4
No	1547	93.6
*Education*		
None completed	49	2.9
Secondary general school	1128	68.2
High School	403	24.4
College/University	74	4.5

The average score on the OSSS-3 was 9.98, with a standard deviation (SD) of 2.14. The median score was 10, and individual scores ranged from 3 to 14, encompassing the full spectrum of the OSSS-3. The average score fell within the 'moderate social support' category, which was also the most common category, with a prevalence of 48.5% (Table 2).

**Table 2:** Frequency distribution of social support in the general population

Social Support	*n*	%
Poor (OSSS = 3–8)	430	26
Moderate (OSSS = 9–11)	802	48.5
Strong (OSSS = 12–14)	422	25.5

ANOVAs were performed to determine whether there were effects of sex or age on the OSSS-3 score (Table 3). No significant effect of gender on the level of social support was found.

**Table 3:** Social Support—mean scores as a function of sex and age

Variables	*n*	OSSS M (SD)	Group differences	Effect size (Eta-squared)
Sex			*p* = 0.086	
Male	738	10.08 (2.13)		0.002
Female	916	9.90 (2.15)		
Age			*p* = 0.008	
60–64	370	9.87 (2.19)		0.008
65–69	465	10.15 (2.10)		
70–74	340	10.20 (2.15)		
75–79	263	9.67 (2.22)		
80–85	216	9.85 (2.15)		

The age of the participants yielded a small, but significant effect (η^2^ = 0.008) on the level of social support. Tukey's honest significant difference test was conducted as a follow-up post hoc test to further examine differences between age groups. Significant differences were found between the 65–69 years and 75–79 years age groups (*p* = 0.027), as well as between the 70–74 years and 75–79 years age groups (*p* = 0.022). Visual inspection of the data revealed an inverted U-shaped distribution, with social support levels continuously decreasing until the 75–79 age group and than increasing slightly by the oldest age group (80–85). However, there was not a statistically significant difference between the 75–79 and 80–85 age groups.

### Internal consistency

The internal consistency of the OSSS-3 within the sample, measured with Cronbach’s alpha, was α = 0.598, whereas McDonald’s omega was ω = 0.632. These relatively low values could be attributed to the low number of items—only three. Brief scales often cannot achieve high levels of Cronbach’s alpha because this measure is strongly dependent on the scale’s length and tends to rise with increasing scale length (Ziegler et al. 2014). For a full description of the items’ characteristics, see Table 4.

**Table 4:** Characteristics of the OSSS-3 items

Item	Mean score (SD)	Item Difficulty Index	Cronbach’s Alpha if Item is Deleted	Skewness	Kurtosis
Close Network	2.64 (0.73)	0.88	0.56	0.31	− 0.58
Concern/Interest of others	3.70 (1.12)	0.93	0.41	-0.61	− 0.84
Neighbors	3.64 (0.99)	0.91	0.49	-0.46	− 0.92

### Construct validity

EFA via principal component analysis (PCA) was performed on the first half of the dataset. A requirement for PCA is a substantial correlation among the items (Bühner 2021). The correlation in our dataset was within the critical range from *r* = 0.30–*r* = 0.90, with an average correlation of *r* = 0.319. (Table 5). As a test for singularity and multicollinearity, the determinant of the matrix should be above the critical threshold of 0.000001 (Field 2018). In our case, the determinant was 0.75.

**Table 5:** Correlation matrix of the OSSS items

Items	Close network	Concern of others	Neighbors
Concern of others	0.355**a		
Neighbors	0.266**	0.392**	

**p < 0.01; a Pearson correlation coefficient between the items

Principal component analysis (PCA) yielded a clear single-factor solution for OSSS-3 (Table 6). This factor exhibited an eigenvalue of 1.640, accounting for 54.68% of the overall variance. The factor loadings of all three items, as displayed in Table 6, consistently demonstrated high values**.** Moreover, the visual inspection of the scree plot concurred with the one-factor solution. A clear turning point in the plot further supported the findings based on the eigenvalue rule. A PA was conducted to determine the number of factors in the OSSS-3. This method involves extracting eigenvalues from 1000 randomly generated datasets designed to match the structure of the original data (3 items and 1655 cases). Factors should only be retained in the actual data if their eigenvalues are greater than those of the random data (O’Connor 2000). Only the first factor in the real data showed an eigenvalue (1.64) greater than the average eigenvalue of the simulated data (1.03). This finding aligns with the eigenvalue criterion, further supporting a one-factor solution for the OSSS-3.

**Table 6:** Factor loadings of the OSSS-3 items

Items	Factor loadings on Component 1	Communalities after extraction
Close Network	0.71	0.50
Concern and interest of others	0.78	0.62
Neighbors	0.72	0.52
Eigenvalue	1.64	
% of variance	54.68	

CFA was computed via AMOS to test the goodness of fit of the one-factor model. The factor loadings of Item 1 and Item 3 had similar values and were set equal to each other. Otherwise, a model cannot be estimated because a model with three estimates is saturated and has exactly one solution.

Factor loadings were assessed for each item. The loading of the third item was slightly below the minimum recommended value of 0.50 (0.446), whereas items one and two had sufficient factor loadings, at 0.565 and 0.683, respectively (Hair et al. 2013).

The model fit measures were used to assess the model’s overall goodness of fit (GFI, CFI, TLI, SRMR and RMSEA), and all values were within their respective common acceptance levels (Bentler 1990; Hair et al. 2013; Hu and Bentler 1998). Overall, the one-factor model for OSSS-3 yielded a good fit to the data (Table 7).

**Table 7:** Model fit measures—recommended range and obtained values

Fit indices	Recommended value	References	Obtained value
GFI	> 0.90	(Hair et al. 2013)	0.99
CFI	> 0.90	(Bentler 1990)	0.98
TLI	> 0.90	(Bentler 1990)	0.94
SRMR	< 0.08	(Hu & Bentler 1998)	0.03
RMSEA	< 0.08	(Hu & Bentler 1998)	0.08

To measure the divergent validity of the OSSS-3, the Pearson correlation coefficients (r) for the PHQ-9, PHQ-15, PDS-5 and SF-12 were calculated. Weak negative correlations were found between OSSS-3 and the PHQ (*r* = 0.253) and between OSSS-3 and the PHQ-15 (*r* = − 0.234). The correlations with the PDS-5 (*r* = − 0.107) and the SF-12 (*r* = 0.101) were very weak.

### Normative data

Table 8 provides normative data for OSSS-3 categorized by age group. The percentiles from this table can be used to compare an individual subject’s OSSS-3 score with the corresponding scores of a matching age group. In addition Gender-specific normative values can be found in Online resource 1, while normative values for every single item can be found in Online resource 2.

**Table 8:** Normative values of OSSS-3 for adults aged between 60 and 85

Age group	60–85 (Total)	60–64	65–69	70–74	75–79	80–85
*N*	1654	370	465	340	263	216
*M*	9.98	9.87	10.15	10.2	9.67	9.85
*SD*	2.15	2.12	2.10	2.15	2.22	2.15
Sum score	Percentile					
3	0.1	0	0	0	0.2	0.4
4	0.4	0.2	0.4	0.1	0.9	0.7
5	1.6	1.1	1.6	0.9	2.8	1.7
6	4.7	4.3	3.8	4.9	6.3	5.3
7	16.7	18.2	14.6	13.4	21.1	18.0
8	26.0	28.1	22.8	21.7	32.5	27.9
9	38.4	41.3	33.7	35.9	42.0	42.7
10	55.4	57.6	52.6	53.3	60.2	55.3
11	74.5	76.2	71.3	71.7	80.6	75.1
12	88.0	89.2	87.3	84.6	90.6	89.8
13	96.1	96.7	96.4	93.8	96.1	98.3
14	100.0	100.0	100.0	100.0	100.0	100.0

### General discussion

The current study provides normative data for the levels of perceived social support measured with the OSSS-3 for the German population aged between 60 and 85 years. The percentile norms allow the direct comparison of individual scores on the OSSS-3 to a corresponding reference sample. The psychometric properties of the OSSS-3 were also explored within the current dataset.

The one-factor solution for the OSSS-3 found by the EFA is in accordance with the results reported by Kocalevent et al. (2018) regarding the general German population. The following CFA was in line with the results from the EFA and confirmed that the one-factor model was a good fit to the data. The internal consistency of the scale, measured with Cronbach’s alpha and McDonald’s omega, is slightly under the widely accepted threshold of 0.7, but is still acceptable considering the briefness of the scale and the multidimensionality of social support as a construct. Divergent validity was also explored and only weak to very weak correlation negative correlation with the PHQ-9 (a depressive symptoms measurement scale), PHQ-15 (a measurement of somatic symptoms), and PDS-5 (PTSD symptom measurement), and SF-12 (a measure of quality of life) was found. This was expected because the measured constructs were only weakly related to the levels of perceived social support. Although these results demonstrate good discriminant validity, the lack of a suitable comparison scale in the current dataset prevents the assessment of convergent validity and limits the ability to fully establish the external validity of the OSSS-3. Overall, the current findings indicate that the scale possesses good psychometric properties for assessing perceived social support in older adults between 60 and 85 years of age, as it was also found to be reliable and with good factor validity.

Our findings revealed that 26% of older adults in our sample reported low levels of perceived social support. This is a concerning proportion, indicating that more than one in four older individuals in Germany perceive insufficient practical or emotional assistance from people around them—be it family members, friends or neighbors—when needed. Compared to data from the general adult population, this proportion is higher, suggesting that older adults may be at increased risk of a perceived lack of social support (Kocalevent et al. 2018).

No significant effect of gender on the level of perceived social support was found, which matches the findings of Kocalevent et al. (2018), who studied perceived social support measured with the OSSS-3 in the general population. However, both of these findings contradict previous studies that reported that women experience higher levels of social support than men do, and the theoretical framework suggests that men and women perceive social support differently because of their respective sex roles (Lozano-Hernández et al. 2022).

One of the main aims of this study was to provide detailed age-normative values for perceived social support in older adults. Our analyses revealed a small but significant age effect on perceived social support (η^2^ = 0.008, *p* = 0.008). Post hoc tests showed significant differences between the 65–69 and 75–79 groups (*p* = 0.027), and between 70–74 and 75–79 (*p* = 0.022). While the 80–85 group did not differ significantly from 75 to 79, an inverted U-shaped pattern emerged: support peaked at ages 65–74, dipped at 75–79 and rose slightly again at 80–85. Such trends would likely be masked by broader age groupings, as often used in general population norms, which can overlook the diversity of aging trajectories.

A possible explanation for the observed inverted U-shaped distribution of perceived social support in older adults is survival bias. Specifically, older adults reporting very low levels of perceived social support may be at a higher risk of mortality, leading to their underrepresentation in the oldest age groups. Consequently, those who survive into advanced age tend to report higher levels of perceived social support. This selective survival could produce the appearance of an inverted U-shaped distribution rather than the continuous decline in social support with increasing age reported by Kocalevent et al. (2018) in their normative data for the general population. This discrepancy further highlights the importance of developing finer-grained normative values specifically tailored for older adults to more accurately capture possible age-related variations in social support.

With the expected continuation of population aging, the number of older adults experiencing low levels of perceived social support could rise further. Divorce rates are rising, and as a consequence, more older adults in the future could be living alone, which is a risk factor for loneliness (Teater et al. 2021). Additionally, more of the next generations of older adults are childless (Kreyenfeld 2018). Research has shown that childless older adults tend to have significantly fewer social interactions (Baranowska-Rataj & Abramowska-Kmon 2019).

The data from the current study are derived from the German older population and are in line with the assumption that many older people in the West are suffering from low levels of perceived social support. However, social support is affected by the cultural context. For example, in contrast to Western society, which is mostly individualistic (Cohen et al. 2016), East Asian culture emphasizes the collective welfare and prosperity of intergenerational families (Chen 2013). One might expect that in East Asian culture, older adults will be provided with higher levels of perceived social support. However, recent studies have shown that ageism in Eastern societies is now prevalent to a similar degree as in the West (Hövermann & Messner 2023; North 2022). This challenges the assumption that older adults in these regions have universally higher levels of perceived social support, suggesting that the risk of low levels of perceived social support may be a global issue for older adults. 

The worrying low levels of perceived social support found in the current study and the possibility of an even wider spread of loneliness and social isolation among older adults in the future emphasize the importance of detailed normative values for the levels of perceived social support for older adults. This problem should be further addressed, and possible solutions should be further sought, as the social and financial costs of the social isolation of the older generation can be very high (Shaw et al. 2017). For example, engineers are already working toward a possible solution and are developing AI-driven social robots for older adults (Breazeal et al. 2019). For a comprehensive review of interventions for loneliness and isolation among older adults, see (Fakoya et al. 2020).

The main limitation of the current study is that it did not examine the associations between the perceived social support measured with the OSSS-3 and other lengthy and well-established instruments for assessing social support such as the Duke Social Support Index (Koenig et al. 1993). This could allow a better assessment of the external validity of the scale.

Future studies should also examine the semantic meaning of the third item of OSSS-3 in greater detail, as it specifically refers to how easy it is to obtain practical help from neighbors when needed. This narrow focus may overlook other important sources of social support, potentially leading to lower scores—even when practical help is readily available from friends or family. This consideration may be valuable in future adaptations or refinements of the scale. This potential shortcoming further highlights the importance of comparing the OSSS-3 with a longer, well-established instrument for assessing perceived social support. 

Cross-sectional data cannot disentangle age effects from cohort effects. While normative values are still valuable for practical use, future research should apply longitudinal designs to separate between the two effects.

As the data were collected in 2008, norms derived from a more recent data sample can further build upon the current findings. Representative studies are rare and costly, but remain the gold standard for developing normative values. To our knowledge, no other studies provide normative data specifically for older adults. Thus, while these norms may not fully reflect current social dynamics, they are valuable and preferable to having no norms at all. The test–retest validity of the OSSS-3 has yet to be explored and could allow us to assess the consistency of the scale better. Considering the increase in the number of very old adults (over 85 years of age), detailed normative values for this age group also increase in significance.

## Conclusion

The OSSS-3 is a brief and economic instrument with good psychometric properties that is particularly well suited for large-scale projects. The provided norm values for older adults could be a useful tool for comparing individual results on the OSSS-3 to those of an age-matched reference sample. The low levels of perceived social support reported by approximately 20% of the older adults in our sample are worrying. The expected continuation of population aging, among other sociological factors discussed, emphasizes the importance of continuing empirical research on this topic.

## Competing Interests

The authors declare no competing interests.

## Supplementary Information

Below is the link to the electronic supplementary material.

Supplementary file1 (DOCX 10 KB)

Supplementary file2 (DOCX 11 KB)

## Data Availability

No datasets were generated or analysed during the current study.
