# Supplementary material for: Normative values and psychometric properties of the Oslo Social Support Scale-3 (OSSS-3) for adults aged 60 to 85 years
Source: Eur J Ageing. 2025 Jul 8;22(1):32. doi: 10.1007/s10433-025-00867-9 (PMC12238707; doi:10.1007/s10433-025-00867-9)
Supplement: Supplementary file 1 — Supplementary file1 (DOCX 10 KB) [file 10433_2025_867_MOESM1_ESM.docx]

*Gender-specific normative values of OSSS-3 for adults aged between 60 and 85*

|  | **Total** | **Men** | | | | | **Women** | | | | |
| --- | --- | --- | --- | --- | --- | --- | --- | --- | --- | --- | --- |
| Age group | **60–85** | **60-64** | **65-69** | **70-74** | **75-79** | **80-85** | **60-64** | **65-69** | **70-74** | **75-79** | **80-85** |
| *N* | **1654** | **182** | **224** | **156** | **109** | **68** | **188** | **241** | **184** | **154** | **148** |
| *M* |  | 9.93 | 10.18 | 10.28 | 9.95 | 9.91 | 10.13 | 10.66 | 10.12 | 9.46 | 9.82 |
| *(SD)* |  | 1.96 | 2.09 | 2.22 | 2.33 | 2.17 | 2.11 | 2.09 | 2.08 | 2.10 | 2.15 |
| Sum score | Percentile | | | | | | | | | | |
| 3 | .1 | 0.0 | 0.0 | 0.0 | .5 | 0.0 | 0.0 | 0.0 | 0.0 | 0.0 | .5 |
| 4 | .4 | 0.0 | .5 | .2 | 1.3 | 0.0 | .3 | .4 | 0.0 | .6 | 1.1 |
| 5 | 1.6 | 1.1 | 1.1 | 1.3 | 4.8 | 1.8 | 1.1 | 2.1 | .5 | 1.4 | 1.6 |
| 6 | 4.7 | 3.9 | 3.7 | 5.9 | 6.0 | 4.8 | 4.7 | 4.0 | 4.0 | 6.5 | 5.5 |
| 7 | 16.7 | 15.2 | 15.0 | 13.7 | 16.2 | 20.1 | 21.0 | 14.2 | 13.1 | 24.7 | 17.1 |
| 8 | 26.0 | 24.3 | 21.6 | 21.4 | 24.7 | 28.6 | 31.7 | 23.9 | 22.0 | 38.1 | 27.6 |
| 9 | 38.4 | 36.2 | 31.0 | 35.1 | 40.6 | 41.4 | 46.3 | 36.3 | 36.6 | 43.0 | 43.3 |
| 10 | 55.4 | 58.0 | 53.1 | 48.6 | 55.7 | 49.8 | 57.2 | 52.1 | 57.3 | 63.4 | 57.8 |
| 11 | 74.5 | 77.9 | 73.5 | 68.2 | 76.3 | 72.5 | 74.4 | 69.3 | 74.8 | 83.7 | 76.3 |
| 12 | 88.0 | 91.3 | 88.5 | 83.9 | 87.0 | 91.8 | 87.2 | 86.2 | 85.2 | 93.1 | 88.9 |
| 13 | 96.1 | 98.7 | 94.0 | 93.3 | 91.4 | 97.9 | 94.7 | 98.6 | 94.3 | 99.4 | 98.5 |
| 14 | 100.0 | 100.0 | 100.0 | 100.0 | 100.0 | 100.0 | 100.0 | 100.0 | 100.0 | 100.0 | 100.0 |
