# Supplementary material for: Normative values and psychometric properties of the Oslo Social Support Scale-3 (OSSS-3) for adults aged 60 to 85 years
Source: Eur J Ageing. 2025 Jul 8;22(1):32. doi: 10.1007/s10433-025-00867-9 (PMC12238707; doi:10.1007/s10433-025-00867-9)
Supplement: Supplementary file 2 — Supplementary file2 (DOCX 11 KB) [file 10433_2025_867_MOESM2_ESM.docx]

*Normative values for adults aged between 60 and 85* *of Oslo 1: How many people are so close to you that you can count on them if you have great personal problems?*

| Age group | **60–85**  **(Total)** | **60-64** | **65-69** | **70-74** | **75-79** | **80-85** |
| --- | --- | --- | --- | --- | --- | --- |
| *N* | **1654** | **370** | **465** | **340** | **263** | **216** |
| *M* | 2.64 | 2.67 | 2.68 | 2.72 | 2.55 | 2.49 |
| *SD* | .73 | .71 | .74 | .72 | .76 | .72 |
| Score | Percentile | | | | | |
| 1 | 2.4 | 1.7 | 2.2 | 1.3 | 3.6 | 4.4 |
| 2 | 46.6 | 43.7 | 44.3 | 41.3 | 54.8 | 55.3 |
| 3 | 87.0 | 87.6 | 85.7 | 85.6 | 87.0 | 91.3 |
| 4 | 100.0 | 100.0 | 100.0 | 100.0 | 100.0 | 100.0 |

*Normative values for adults aged between 60 and 85* *of Oslo 2: How much interest and concern do people show in what you do?*

| Age group | **60–85**  **(Total)** | **60-64** | **65-69** | **70-74** | **75-79** | **80-85** |
| --- | --- | --- | --- | --- | --- | --- |
| *N* | **1654** | **370** | **465** | **340** | **263** | **216** |
| *M* | 3.70 | 3.62 | 3.77 | 3.79 | 3.60 | 3.69 |
| *SD* | 1.12 | 1.13 | 1.12 | 1.10 | 1.15 | 1.11 |
| Score | Percentile | | | | | |
| 1 | 1.4 | 1.1 | 1.5 | 1.0 | 1.4 | 2.5 |
| 2 | 24.1 | 26.8 | 22.4 | 21.9 | 27.7 | 22.5 |
| 3 | 29.6 | 32.7 | 27.0 | 27.0 | 33.3 | 29.5 |
| 4 | 74.6 | 77.7 | 71.8 | 71.6 | 77.0 | 77.4 |
| 5 | 100.0 | 100.0 | 100.0 | 100.0 | 100.0 | 100.0 |

*Normative values for adults aged between 60 and 85 of* Oslo 3: How easy is it to get practical help from neighbours if you should need it?

| Age group | **60–85**  **(Total)** | **60-64** | **65-69** | **70-74** | **75-79** | **80-85** |
| --- | --- | --- | --- | --- | --- | --- |
| *N* | **1654** | **370** | **465** | **340** | **263** | **216** |
| *M* | 3.64 | 3.59 | 3.70 | 3.69 | 3.51 | 3.68 |
| *SD* | .99 | .98 | .97 | .99 | 1.00 | 1.00 |
| Score | Percentile | | | | | |
| 1 | 2.9 | 2.6 | 2.2 | 2.7 | 3.8 | 4.0 |
| 2 | 11.0 | 12.4 | 9.0 | 11.5 | 13.6 | 8.8 |
| 3 | 42.4 | 44.9 | 41.3 | 38.0 | 47.4 | 41.7 |
| 4 | 79.6 | 81.6 | 77.3 | 78.5 | 84.1 | 77.9 |
| 5 | 100.0 | 100.0 | 100.0 | 100.0 | 100.0 | 100.0 |
